# Clinical efficacy of inhaled corticosteroids in patients with coronavirus disease 2019: A living review and meta-analysis

**DOI:** 10.1371/journal.pone.0294872

**Published:** 2023-11-28

**Authors:** Su-Yeon Yu, Miyoung Choi, Seungeun Ryoo, Chelim Cheong, Kyungmin Huh, Young Kyung Yoon, Su Jin Jeong

**Affiliations:** 1 Department of Medical Information, College of Nursing and Health, Kongju National University, Gongju, Republic of Korea; 2 Division for Healthcare Technology Assessment Research, National Evidence-based Healthcare Collaborating Agency, Seoul, Republic of Korea; 3 Department of Public Health, Korea University Graduate School, Seoul, Korea; 4 Health-Care Insight Research, Seoul, Republic of Korea; 5 Division of Infectious Diseases, Department of Medicine, Samsung Medical Center, Sungkyunkwan University School of Medicine, Seoul, Republic of Korea; 6 Division of Infectious Diseases, Department of Internal Medicine, Korea University College of Medicine, Seoul, Republic of Korea; 7 Division of Infectious Disease, Department of Internal Medicine, Severance Hospital, Yonsei University College of Medicine, Seoul, Republic of Korea; Keele University & University Hospitals of North Midlands (UHNM) NHS Trust, UNITED KINGDOM

## Abstract

Inhaled corticosteroids are known to be relatively safe for long-term use in inflammatory respiratory diseases and it has been repurposed as one of the potential therapies for outpatients with coronavirus disease 2019 (COVID-19). However, inhaled corticosteroids have not been accepted for COVID-19 as a standard therapy because of its lack of proven benefits. Therefore, this study aimed to evaluate the effectiveness of inhaled corticosteroids in patients with COVID-19. Randomized controlled trials comparing the efficacy of inhaled corticosteroid treatment in patients with COVID-19 were identified through literature electronic database searches up to March 10, 2023. Meta-analyses were conducted for predefined outcomes, and the certainty of evidence was graded using the grading of recommendations, assessment, development, and evaluation approach. Overall, seven trials (eight articles) were included in this systematic review. Compared with usual care, inhaled corticosteroids was associated with significantly improved clinical recovery at 7 and 14 days in patients with COVID-19. In subgroup analysis, only budesonide showed significant efficacy in clinical recovery, whereas no significant benefit was observed for ciclesonide. Moreover, inhaled corticosteroids use was not significantly associated with all-cause hospitalization, all-cause mortality, admission to intensive care unit, or the use of mechanical ventilation. Our systematic review used evidence with very low to moderate certainty. Although based on limited evidence, our results suggest that inhaled corticosteroids treatment, especially budesonide, improves the clinical recovery of patients with COVID-19. More trials and meta-analyses are needed to assess the efficacy of inhaled corticosteroids for COVID-19 treatment.

## Introduction

Coronavirus disease 2019 (COVID-19), caused by the severe acute respiratory syndrome coronavirus 2 (SARS-CoV-2), was first reported in China in late 2019 [1]. Most infected patients are asymptomatic and show only mild symptoms; however, the remaining patients experience a severe form of the disease. Several potential therapeutics have been proposed in the early phase of the COVID-19 pandemic, such as hydroxychloroquine, lopinavir/ritonavir, and convalescent plasma transfusion; however, other agents, including corticosteroids, have been known to improve clinical outcomes [2]. Corticosteroids have been widely used in hospitalized patients with COVID-19 who are in need of oxygen therapy, and several studies have demonstrated the beneficial effects of corticosteroid use [2, 3].

Corticosteroids have been considered as potential therapeutic drugs because their early use has been shown to reduce the systemic inflammatory response and accelerate recovery from pulmonary infection [4]. This may be related to the role of glucocorticoids, which inhibit cytokine synthesis and reduce the proliferation and regulation of T cells and macrophages [5]. However, the systemic use of corticosteroids can theoretically cause several side effects [6] and does not inhibit secondary bacterial infections or viral clearance [7]. Inhaled corticosteroids (ICS) are essential for treating major respiratory diseases, such as asthma and chronic obstructive pulmonary disease (COPD) [8]. ICS are also known to be beneficial in COVID-19 treatment, as they reduce the expression of key proteins that promote the entry of the virus into host cells and downregulate COVID-19-related genes [9, 10]. The safety of ICS has been extensively studied since their introduction for the treatment of asthma and COPD over 20 years ago [11, 12]. There have been fewer studies performed on ICS use for COVID-19 treatment compared to systemic corticosteroid use. This study aimed to systematically review the clinical efficacy of ICS among patients with COVID-19.

## Methods

We conducted a systematic review and a series of meta-analyses of randomized clinical trials (RCTs) in accordance with the recommendations of the Cochrane Handbook and the Preferred Reporting Items for Systematic Review and Meta-Analysis statement [13]. The protocol for this review was prospectively registered in the International Prospective Register of Systematic Reviews under the registration number CRD42022382250.

### Search strategy

A literature search was initially conducted to include RCTs regarding ICS therapy for COVID-19 infection initially on June 11, 2021, and the search was updated every month through March 10, 2023. The sources included PubMed, Ovid-EMBASE, CENTRAL, and the Korean database, KMBASE. The key terms included in the search were “COVID-19,” “SARS-CoV-2,” and ([“inhaled” OR “inhalant”] AND [“glucocorticoids” OR “steroid” OR “corticosteroid”]), “budesonide,” “ciclesonide,” and others. A manual search of the reference lists of the included articles and review articles was performed to identify related studies. A complete electronic search strategy for each database is presented in S1 Table.

### Eligibility criteria and study selection

We included studies that 1) recruited adults with COVID-19, 2) used ICS as an intervention, 3) controlled for placebo or standard of care treatment, 4) collected outcomes including clinical recovery and hospital admission, 5) were written in English or Korean, and 6) were designed as RCTs. Two review authors (SY and SJ) separately evaluated publications for inclusion based on the title and abstract and subsequently reviewed the relevant full-text articles. Disagreements between the reviewers were resolved by consensus with the involvement of a third independent reviewer (MC).

### Risk of bias assessment and data extraction

Two authors (SY and CC) independently assessed the quality of the selected studies using the Cochrane risk of bias tool [14]. Disagreements were addressed by consensus, with the participation of a third review author (MC or SJ).

Two authors (CC and SR) separately extracted information from each included trial and checked for accuracy through discussions and the third reviewer (MC)’s opinion. The following information was included in the data extraction form: first author, trial name, publication date, enrollment period, study site by country, setting where the patients were enrolled, outcomes collected, characteristics of study participants, ingredients, dose and duration of ICS therapy, type of comparator, and outcomes. The primary outcomes were clinical recovery and all-cause hospitalization at 28 days. Clinical recovery was defined as the alleviation of all COVID-19–related symptoms by 7 or 14 days according to the definition of included studies. Secondary outcomes included all-cause mortality at 28 days, admission to the intensive care unit (ICU), and the use of mechanical ventilation. Some data were collected from the supplementary materials or by using the intention-to-treat principle. We planned to explore publication bias using funnel plots for outcomes for which data from 10 or more studies were available.

### Rating the certainty of evidence

Certainty of evidence was graded using the grading of recommendations, assessment, development, and evaluations (GRADE) approach for outcomes [15]. The GRADE approach includes following factors for certainty assessment, such as risk of bias, inconsistency, indirectness, imprecision, and publication bias etc. The certainty of evidence presented as high, moderate, low, or very low quality based on considerations of all factors.

### Data synthesis and statistical analysis

For each included trial, dichotomous outcomes were presented as risk ratios (RR) with a random-effects model (Mantel–Haenszel method), and 95% confidence intervals (CIs), and continuous outcomes (time to clinical recovery) were presented as mean differences or hazard ratios (HR) with a random-effects model (inverse-variance method). When the participants included in each study vary in their demographic and clinical characteristics, the random effects model estimates are known to be more conservative. The Higgins I^2^ test was used to determine the level of heterogeneity within studies. Statistical analyses were performed using Review Manager Software version 5.4 (the Cochrane Collaboration, 2020) and R Software version 4.2.1 (R Core Team, 2021).

## Results

### Description of included studies

**Fig 1:**
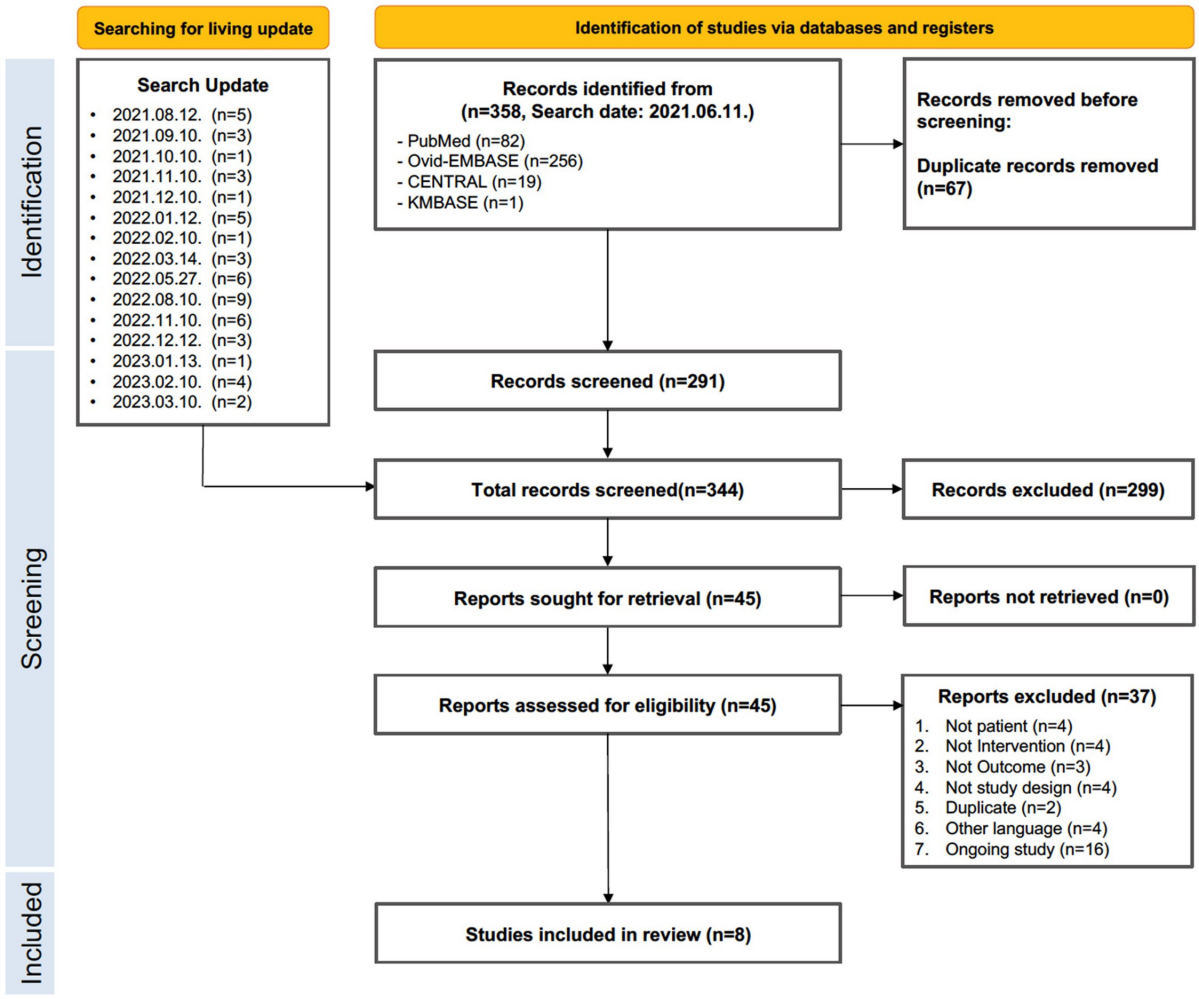
Preferred reporting items for systematic reviews and meta-analyses study flowchart.

In total, 358 articles were retrieved from the databases, resulting in 291 articles after excluding duplicates. Based on the selection criteria, 45 articles were selected for full-text review. Of these, eight studies were included in this systematic review. Patient enrollment occurred at various times in the included studies, spanning from April 2020 to July 2021. All the included articles were performed prior to the emergence of the Delta variant of COVID-19. Details of the study selection and a flowchart of the review are shown in Fig 1. Of the eight studies, two used budesonide [16, 17] and six used ciclesonide [18–23].

The baseline characteristics of the included studies are presented in Table 1.

**Table 1:** Baseline study characteristics of randomized controlled trials on inhaled corticosteroids included in the analysis.

Trial name Trial number	First author Year	Intervention group (n) ^a^	Control (n)^a^	Country (no. of sites)	Enrollment period	Variants of Concern	Inclusion criteria		Recruitment	Enrollment	Outcome collection	Patient age (years) (mean, median)	Published date
							Symptom	Respiratory status					
STOIC trial^b^ NCT04416399	Ramakrishnan 2021	Budesonide 800 μg twice daily ≤28 days (73)	Usual care (73)	UK	Jul 16 to Dec 9, 2020	Pre-Delta	≤7 days from symptom onset	No criteria	Local primary care networks, local COVID-19 testing sites, or multichannel advertising	Home visit	Telephone or trial center visit	18 + (Budesonide, 44; Control, 46)	Lancet Respir Med Apr 9, 2021
PRINCIPLE trial ISRCTN86534580	Yu 2021	Budesonide 800 μg twice daily for 14 days (833)	Usual care (886)	UK	Nov 27, 2020 to Mar 31, 2021	Pre-Delta	≤14 days from symptom onset Excluding those with much improved and mild symptoms		Study center visit or telephone followed by confirming medical records	General medical practices, online or telephone	Outpatient facility visit, telephone or self-report	60 + or 50 + with comorbidities (Budesonide, 65; Control, 65)	Lancet Sep 4, 2021
RACCO jRCTs031190269	Terada-Hirashima 2022	Ciclesonide 400 μg three times daily for 7 days (41)	Usual care (48)	Japan (22)	Apr 3 to Sep 18, 2020	Pre-Delta	Asymptomatic or mildly ill	No signs of pneumonia on chest X-ray	Study centers	Hospital	Hospital	20 + (60 + Ciclesonide, 12%; Control, 23%)	Drug Discov Ther Nov 20, 2022
NCT04330586	Song 2021	Ciclesonide 320 μg twice daily for 14 days (35)	Usual care (26)	Korea (7)	May 8, 2020 to Mar 31, 2021	Pre-Delta	≤7 days from symptom onset or ≤3 days from diagnosis	National Early Warning Score ranging from 0 to 4 Oxygen saturation ≥95% room air	Study centers^c^	Hospital	Hospital	19 + (Ciclesonide, 45; Control, 49)	J Clin Med Aug 12, 2021
NCT04377711	Clemency 2021	Ciclesonide 320 μg twice daily for 30 days (197)	Placebo (203)	USA (10)	Jun 11 to Nov 3, 2020	Pre-Delta	COVID-19 symptom and ≤3 days from diagnosis	Oxygen saturation ≥93% breathing room air	Study centers	Doctor’s office or home visit	Telephone or outpatient facility visit	12 + (Ciclesonide, 44; Control, 43	JAMA Nov 21, 2021
CONTAIN trial NCT04435795	Ezer 2021	Inhaled ciclesonide (100–800 μg daily) and intranasal ciclesonide (200 μg daily) for 14 days (105)	Placebo (98)	Canada	Sep 15, 2020 to Jun 8, 2021	Pre-Delta	COVID-19 symptom	Excluding those who needed oxygen therapy	Telephone	Telephone or dispensed by mail	Online survey	18 + (Ciclesonide, 35; Control, 35)	BMJ Nov 2, 2021
COVERAGE France trial NCT04356495	Duvignaud 2021	Ciclesonide 320 μg twice daily for 10 days (110)	Vitamin + trace elements (107)	France (14)	Dec 29, 2020 to Jul 23, 2021	Pre-Delta	≤7 days from symptom onset	Excluding those who needed acute oxygen therapy	Study centers	Outpatient facility or home visit	Outpatient facility visits and (mainly) telephone	60 + or 50 + with risk factors (Ciclesonide, 62; Control, 63)	Clin Microbiol Infect Mar 16, 2022
HALT COVID-19 trial NCT04381364	Brodin 2023	Ciclesonide 320 μg twice daily for 14 days (48)	Standard care (51)	Sweden (9)	Jun 1, 2020 to May 17, 2021	Pre-Delta	Receiving oxygen therapy for ≤48 hours		Study centers	Hospital	Hospital	Age range: 18 + Ciclesonide 61 Control 59	BMJ Open Feb 22, 2023

^a^Numbers included in meta-analysis

^b^Including those without confirmed COVID-19 (6%)

^c^All patients with COVID-19 in Korea were hospitalized or admitted to community treatment centers during the study period.

Abbreviations: COVID-19, coronavirus disease 2019.

The results of the risk-of-bias summary are presented in S1 Fig. Two studies involved patients with high risk (≥50 years old with risk factors or ≥60 years old) [17, 20], whereas six studies involved adults with mild symptoms. All patients in the five studies were in outpatient and/or home care settings, whereas three studies in Japan, South Korea, and Sweden recruited inpatients [18, 22, 23]. The participants in the Japanese study were asymptomatic or mildly ill at baseline [18]. Regarding the study from South Korea, all patients with COVID-19 in South Korea were required to be admitted to a hospital or community treatment center during the study period (from May 2020 to March 2021). Disease severity among patients in the Korean study was not different from that in other studies; those with a >5 National Early Warning Score or oxygen saturation <95% of room air were excluded from the study [22]. Two studies included a placebo group [19, 21], whereas in six studies, the patients were compared with a usual-care group. Six studies had a low risk of bias in all the domains such as randomization process, performance bias, attrition bias, and reporting bias. Heterogeneity results were not significantly high however we explored that by subgroup analysis. The GRADE evidence profiles and a summary of the findings are presented in Table 2.

**Table 2:** The GRADE summary of findings table of primary and secondary outcomes.

Outcomes	Anticipated absolute effects (95% CI)		Relative effect (95% CI)	No. of participants (studies)	Certainty of evidence (GRADE)
	Risk with standard of care/placebo	Risk with Neutralizing monoclonal antibody			
Clinical recovery at 14 days	333 per 1,000	402 per 1,000 (359 to 452)	RR 1.21 * (1.08 to 1.36)	2585 (6 RCTs)	⨁⨁⨁◯ Moderate^a^
Clinical recovery at 7 days	182 per 1,000	219 per 1,000 (188 to 252)	RR 1.20 * (1.03 to 1.38)	2591 (6 RCTs)	⨁⨁⨁◯ Moderate^a^
Time to recovery	0	MD 1.42 lower (3.72 lower to 0.89 higher)	-	1720 (3 RCTs)	⨁⨁◯◯ Low^a^^,c^
All-cause hospitalization at 28 days	93 per 1,000	73 per 1,000 (41 to 128)	RR 0.78 (0.44 to 1.37)	2539 (5 RCTs)	⨁◯◯◯ Very low^a^^,^^b^^,^^c^
All-cause mortality at 28 days	9 per 1,000	6 per 1,000 (3 to 15)	RR 0.71 (0.32 to 1.57)	2788 (8 RCTs)	⨁⨁⨁◯ Moderate^c^
Intensive care unit admission	30 per 1,000	17 per 1,000 (9 to 33)	RR 0.57 (0.30 to 1.08)	1648 (2 RCTs)	⨁⨁◯◯ Low^a^^,^^c^
Mechanical ventilation	20 per 1,000	17 per 1,000 (8 to 34)	RR 0.84 (0.41 to 1.68)	1658 (2 RCTs)	⨁⨁⨁◯ Moderate^c^

GRADE Working Group grades of evidence

High certainty: We are confident that the true effect lies close to the estimate of the effect.

Moderate certainty: We are moderately confident in the effect estimate; the true effect is likely to be close to the estimate of the effect; however, there is a possibility that it is substantially different.

The risk in the intervention group (and its 95% CI) is based on the assumed risk in the comparison group and the relative effect of the intervention (and its 95% CI).

^a^Risk of bias downgraded by one level owing to lack of blinding and allocation concealment.

^b^Inconsistency downgraded by one level due to moderate heterogeneity.

^c^Imprecision downgraded by one level due to the low sample size and a wide confidence interval consistent with the possibility of benefit and harm.

**Abbreviations:** GRADE, Grading of Recommendations, Assessment, Development, and Evaluation; CI, confidence interval; RR, risk ratio; RCT, randomized controlled trial; MD, mean difference

### Primary outcomes

**Fig 2:**
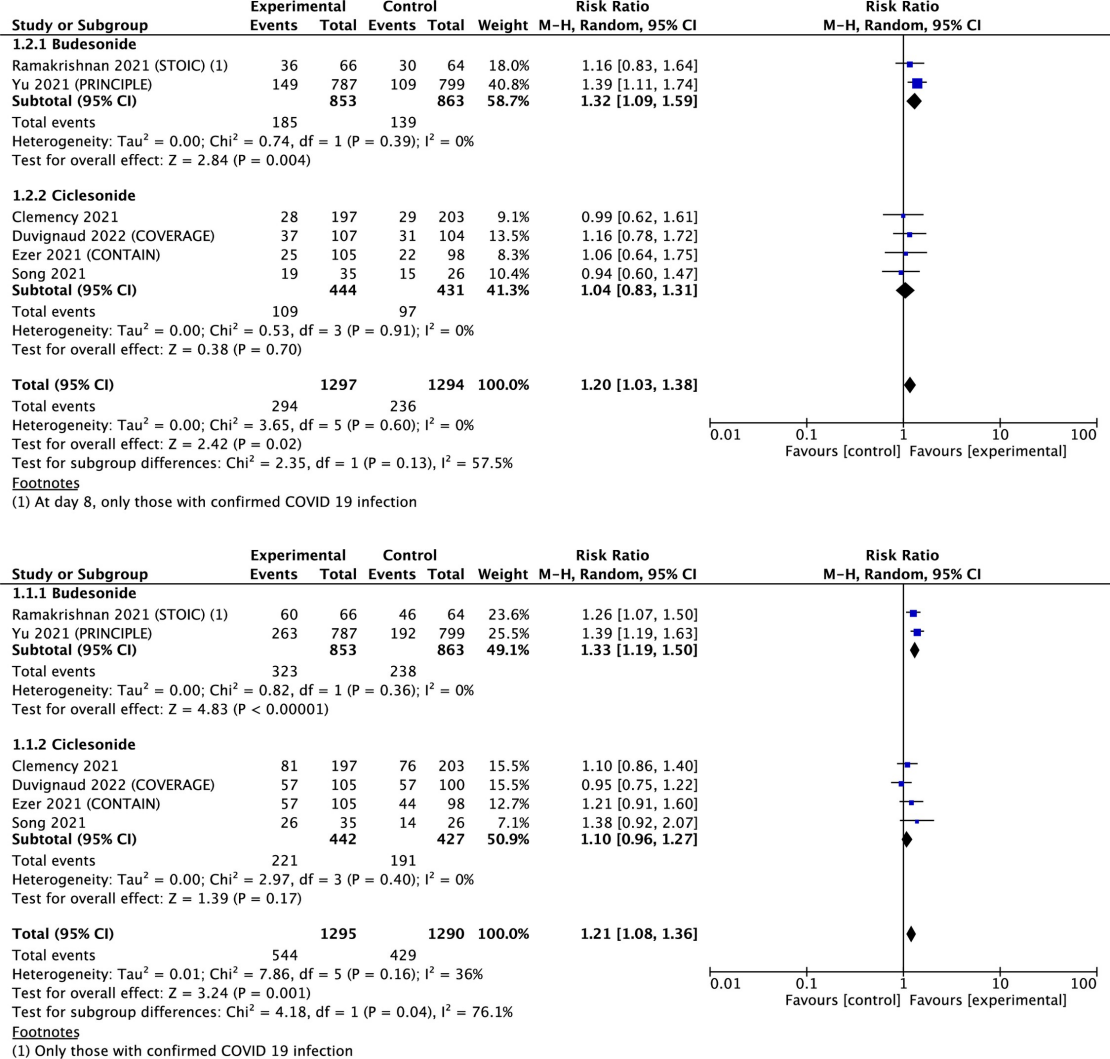
Forest plot of clinical recovery. **(A)** Clinical recovery at 7 days. **(B)** Clinical recovery at 14 days.

Six articles reported clinical recovery after 7 and 14 days of treatment. Compared with the control group, the group treated with ICS showed a significant association with improved clinical recovery at 7 and 14 days (RR 1.20 [95% CI, 1.03–1.38; I2, 0%] and 1.21 [95% CI, 1.08–1.36; I2, 36%], respectively) (Table 2 and Fig 2). In sub-group analysis, compared with placebo or usual care, ciclesonide was not significantly associated with improved clinical recovery, whereas budesonide was significantly associated with improved clinical recovery at 7 and 14 days (RR 1.32 [95% CI, 1.09–1.59; I2, 0%] and 1.33 [95% CI, 1.19–1.50; I2, 0%], respectively) (Table 2 and Fig 2). Subgroup analysis with the types of comparators (placebo and others) was followed by ICS ingredients (S2 Fig). Two studies with budesonide had non-placebo controls, while two placebo studies and two other control studies had ciclesonide. In studies with ciclesonide, no significant effect on clinical recovery at 7 and 14 days was observed in both placebo studies and other control studies.

**Fig 3:**
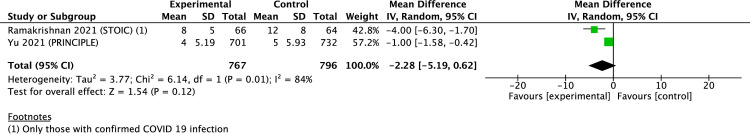
Forest plot of time to clinical recovery (days).

No significant effect on time to clinical recovery was observed with the use of ICS, regardless of whether it was all ICS, budesonide, or ciclesonide (Table 2 and Fig 3).

**Fig 4:**
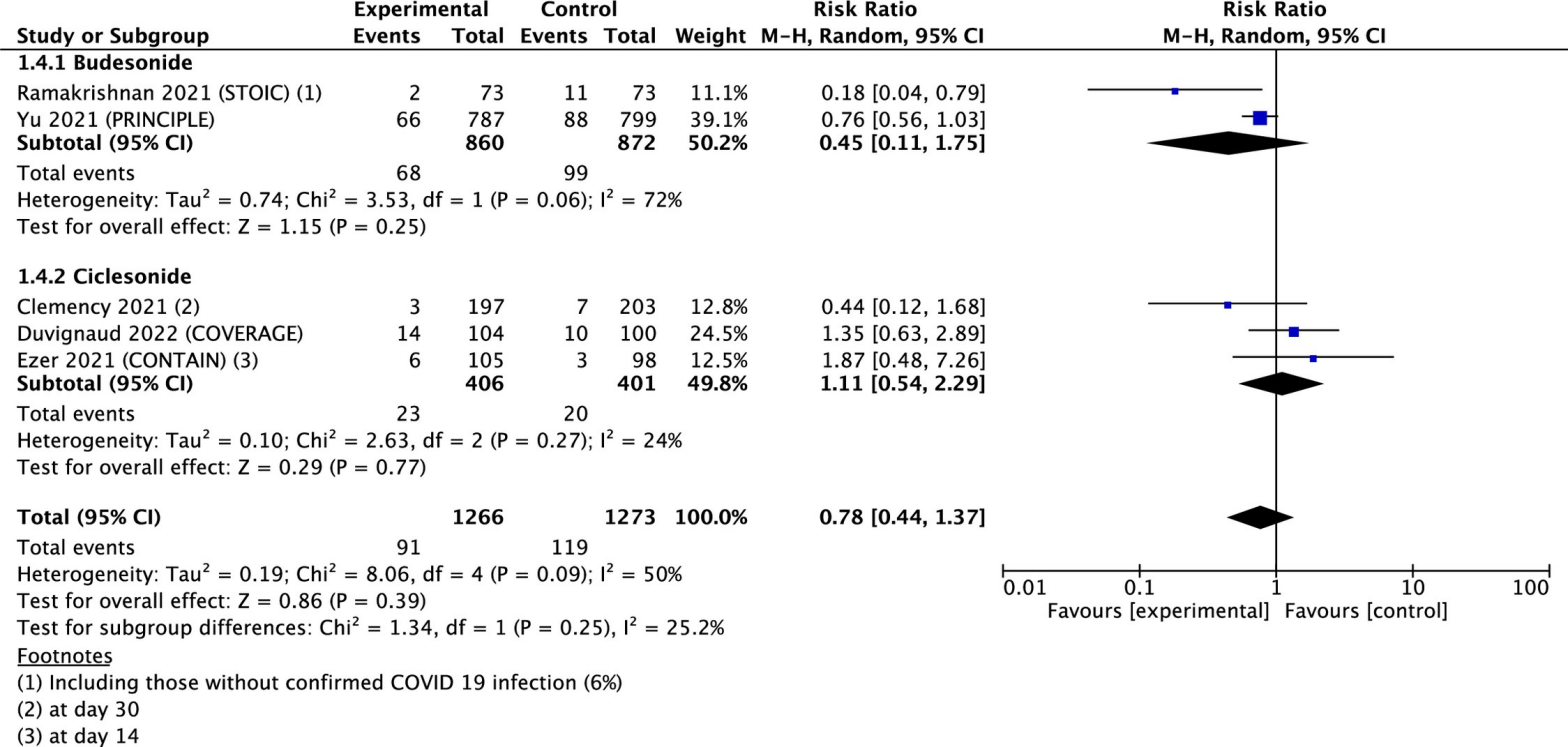
Forest plot of all cause hospitalization at 28 days.

All-cause hospitalization at 28 days was reported in five articles in which the participants were all outpatients. Hospital admission at 14 days was not significantly associated with the use of ICS, regardless of whether it was all ICS, budesonide, or ciclesonide. (Table 2 and Fig 4).

### Secondary outcomes

The secondary outcomes were all-cause mortality at 28 days, ICU admission, and use of mechanical ventilation. All seven articles reported all-cause 28-day mortality; however, death events were reported in only three articles, which included study participants with risk factors [17, 20] (Table 2 and S3 Fig). ICS use was not significantly associated with all-cause mortality. ICU admission and use of mechanical ventilation were reported in one article on ciclesonide [17]. Neither ICU admission nor the use of mechanical ventilation was significantly associated with the use of ciclesonide (Table 2 and S4 and S5 Figs).

## Discussion

The group treated with ICS was significantly associated with improved clinical recovery at 7 and 14 days compared to the control group, with low heterogeneity (I2 = 0%), contrary to some studies [19–22]. However, ICS treatment did not improve mortality or hospitalization in our study. A systematic review of three randomized clinical trials suggested that ICS therapy reduces the risk of hospitalization in patients unvaccinated against COVID-19. However, the ICS treatment had no significant positive effect on mortality [24]. Some studies have shown that ICS treatment has the possibility of reducing hospitalization in patients with COVID-19.

In STOIC (Steroids in COVID-19) trial, adult outpatients with mild, early COVID-19 (within 7 days from symptom onset) treated with ICS (budesonide 800 μg twice daily, for an average of seven days) were compared with usual-care patients. Among the users of inhaled corticosteroids (ICS), a smaller number of patients required urgent medical evaluation or hospitalization (1%) compared to the usual-care group (14%) at 28 days. Another trial consisting of 400 adults and children aged 12 years or above with mild COVID-19 showed that ICS treatment (ciclesonide 320 μg twice daily for 30 days) reduced the combined outcome of emergency department visits or hospital admissions within 30 days when compared to placebo. Only 1% of the ICS treated group required this combined outcome, while the placebo group had a rate of 5.4% [19].

In the PRINCIPLE (Platform Randomized Trial of Treatments in the Community for Epidemic and Pandemic Illnesses), this study found that treatment with inhaled budesonide 800 μg twice daily did not reduce the risk of hospitalization or death at 28 days compared to usual care. The study included outpatients who were either 65 years and older or 50 years and older with risk factors for severe disease and were diagnosed with COVID-19 [17]. However, a benefit in the time to first self-reported recovery, of an estimated 2.94 days, has been shown in the budesonide group. Moreover, the risk of bias and decrease in confidence in the potential benefit of budesonide was heightened by factors such as: including patients with suspected but not confirmed COVID-19, relying on self-reported outcomes in an open-label trial, and enrolling the control group over a longer period than the intervention group.

Cough and fever are among the most common symptoms of COVID-19 and can persist for weeks to months after COVID-19. Post-COVID syndrome or long COVID is comprised of several symptoms, including cough, chronic fatigue, dyspnea, cognitive impairment, and pain. Discrete guidelines on the pharmacological treatment of various manifestations of post-COVID syndrome are still not well defined. Therefore, relieving symptoms, ranging from cough and fatigue to exertional dyspnea or more critical manifestations, early after recovering from COVID-19 is important. In addition, ICS have been advocated for use in intractable post-viral coughing for symptomatic relief [25]. Therefore, it is clinically valuable that ICS provide additional benefits in relieving symptoms in patients with COVID-19.

ICS is commonly used to manage chronic respiratory conditions, such as asthma and COPD. They are highly effective in reducing inflammation and airway hyperresponsiveness, which ultimately lowers the risk of exacerbations [8, 26]. There are four main types of inhaled steroids that are widely accessible: beclometasone, budesonide, fluticasone, and memetasone. Relatively newer ICS include ciclesonide and flunisolide. In our study, two types of ICS, budesonide and ciclesonide, were analyzed, and more clinical benefits were observed in the study on budesonide use.

There have been reports that the use of ICS is associated with respiratory infection [27]. This may delay viral clearance in patients [28]. However, impairment of the immune response was not observed in another study of patients with asthma receiving ICS [29]. Moreover, the risk of respiratory infection may appear when ICS are administered for a long time; therefore, short-term use in early COVID-19 will not increase the risk. In addition, the effect of ICS may differ depending on the type of respiratory infection, the severity of the patient’s respiratory condition, and the physicochemical properties of ICS. A separate study found that fluticasone remains in the airway lumen and mucus for a longer duration due to its poor solubility and permeability across the airway mucosa [30]. Conversely, budesonide has greater solubility and quickly passes through the airways [30]. With approximately 15–28% lung deposition [31], the lung fraction has the therapeutic effect and is absorbed directly into the systemic circulation. Thus, it has a relatively greater systemic impact than that absorbed through the gut because there is no first pass after lung absorption. A meta-analysis of 17 trials involving patients with asthma revealed an increased risk of upper respiratory infection with fluticasone but not budesonide [32]. In addition, another meta-analysis of 25 trials, including patients with COPD [33] demonstrated an increased risk of pneumonia with fluticasone but not budesonide.

The delivery of ciclesonide to the airways as an inactive compound, which is then converted into its active metabolite by esterases, resulting in slightly different pharmacokinetics compared to other ICS. It also has low oral bioavailability and a high clearance rate, potentially decreasing systemic side effects such as adrenal suppression [34, 35]. Although previous studies have reported that ciclesonide has strong antiviral activity against SARS-CoV-2 [36, 37], clinical trials have failed to show a clear therapeutic effect [19–22]. Another study reported that ciclesonide exerts high selection pressure on SARS-CoV-2 in patients with COVID-19 with asthma, and it may drive the emergence of resistant mutants [38]. Although the anti-inflammatory effect of ICS is expected to be more significant than its antiviral effects, further studies are required.

This study has some limitations. First, the included studies had small numbers of patients (except for the PRINCIPLE trial) and relatively heterogeneous methodologies. There were differences among the studies in terms of the type of ICS, dose, treatment duration, and inclusion criteria. However, it is important to note that all the included studies were RCTs, and they were also assessed as having a low risk of bias in methodology. Second, the relatively larger number of patients in the PRINCIPLE trial may have a substantial impact on the conclusions drawn in our meta-analyses. It is important to note that the PRINCIPLE trial is not a placebo-controlled study; its comparator is usual care, which could potentially introduce bias. Therefore, we conducted additional subgroup analysis, but it was difficult to draw a definite conclusion with only two small studies using placebo (S6 Fig). Third, as most of enrolled studies did not provide sufficient data for the safety of ICS, it was not possible to perform a meta-analysis on their safety. However, ICS have few side effects and have been widely used to control chronic inflammation of the respiratory tract. Fourth, the research periods of the included studies differed; thus, it may have been difficult to reflect the effectiveness of ICS treatment against the current variants of SARS-CoV-2.; thus, it may have been difficult to reflect the effectiveness of ICS treatment against the current variants of SARS-CoV-2. Fifth, individual patient data, which would have enabled various subgroup analyses and confounder adjustments, was not available in our study. Therefore, it is imperative to conduct additional research to validate and explore the factors influencing the results in more detail.

Nevertheless, our study has several strengths. First, to our knowledge, this meta-analysis was conducted using a large number of studies on ICS treatment in patients with COVID-19 and up-to-date evidence, including publications until January 2023. Emerging evidence has led to robust conclusions regarding ICS efficacy. Second, we conducted subgroup analyses to stratify the types of ICS and comparators. We found that differences exist in ICS efficacies based on the present evidence, and comparator.

## Conclusion

The results of this meta-analysis revealed the clinical efficacy of ICS treatment compared with usual care. Based on limited evidence, our results suggest that ICS treatment, especially budesonide, improves the clinical recovery of patients with COVID-19. The safety of ICS treatment in patients with COVID-19 has not yet been established; however, ICS are relatively safe and widely available. In addition, short-term ICS use in the early stages of COVID-19 did not increase the risk of pulmonary infections or side effects. Subsequent randomized clinical trials with a larger number of patients, as well as meta-analyses, are needed to determine the usefulness of ICS treatment in improving the clinical outcomes of patients with COVID-19.

## Supporting information

S1 Table Search strategy. (PDF)

S1 Fig Risk of bias. (PDF)

S2 Fig Clinical Recovery by ICS and comparator (A, B).
(PDF)

S3 Fig All-cause mortality at 28 days. (PDF)

S4 Fig ICU admission. (PDF)

S5 Fig The use of mechanical ventilation. (PDF)

S6 Fig Clinical Recovery by comparator (A, B).
(PDF)
