# Supplementary material for: Clinical efficacy of inhaled corticosteroids in patients with coronavirus disease 2019: A living review and meta-analysis
Source: PLoS One. 2023 Nov 28;18(11):e0294872. doi: 10.1371/journal.pone.0294872 (PMC10684004; doi:10.1371/journal.pone.0294872)
Supplement: S1 Fig — (PDF) [file pone.0294872.s002.pdf]

Supplementary material 2. Risk of bias summary

	Random sequence generation (selection bias)	Allocation concealment (selection bias)	Blinding of participants and personnel (performance bias)	Blinding of outcome assessment (detection bias)	Incomplete outcome data (attrition bias)	Selective reporting (reporting bias)
Clemency 2021	+	+	+	+	+	+
Duvignaud 2022 (COVERAGE)	+	+	−	−	+	+
Ezer 2021 (CONTAIN)	+	+	+	+	+	+
Ramakrishnan 2021 (STOIC)	+	−	−	−	+	+
Song 2021	+	−	?	?	+	+
Terada-Hirashima 2022	+	+	?	+	+	+
Yu 2021 (PRINCIPLE)	+	+	−	−	?	+
