# Supplementary figures and images for: Clinical efficacy of inhaled corticosteroids in patients with coronavirus disease 2019: A living review and meta-analysis
Source: PLoS One. 2023 Nov 28;18(11):e0294872. doi: 10.1371/journal.pone.0294872 (PMC10684004; doi:10.1371/journal.pone.0294872)

## S4 all-cause 28-day mortality

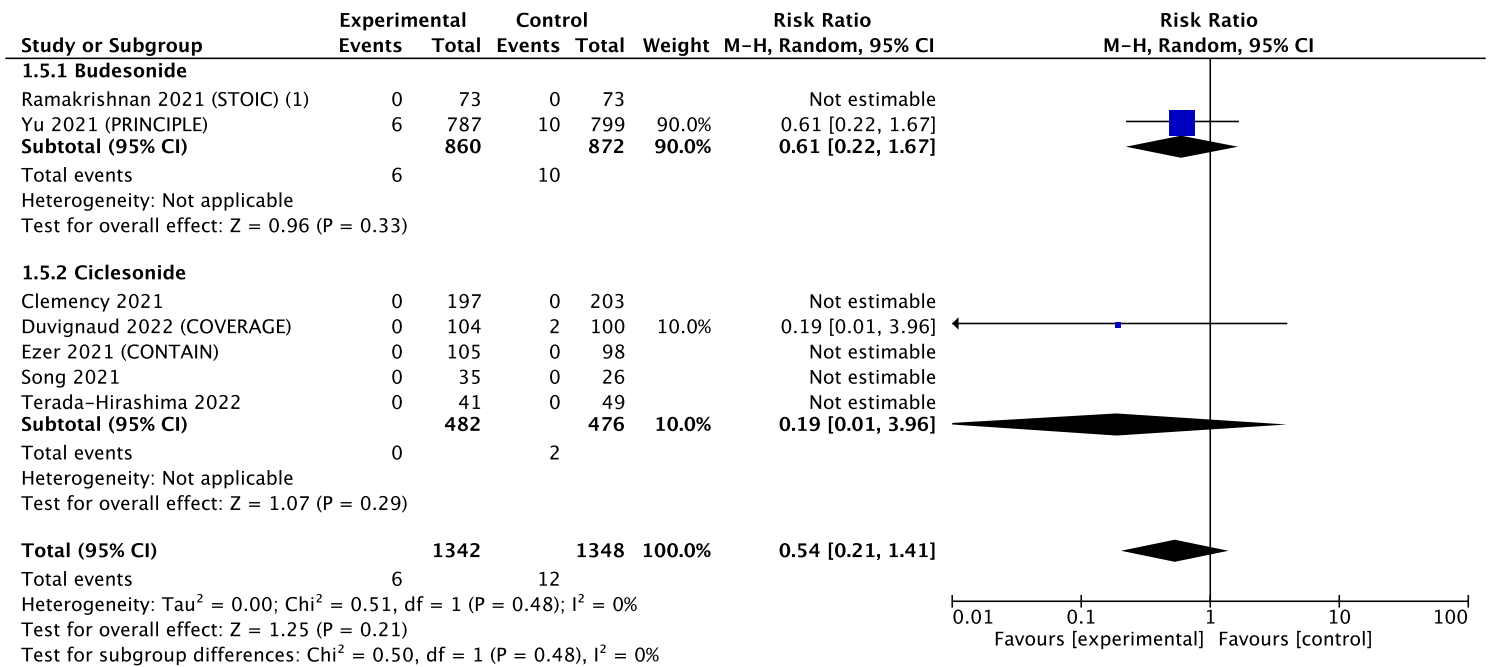

### Footnotes

(1) Only those with confirmed COVID 19 infection

Supplement: S3 Fig — (PDF) [file pone.0294872.s004.pdf]

S5 ICU admission

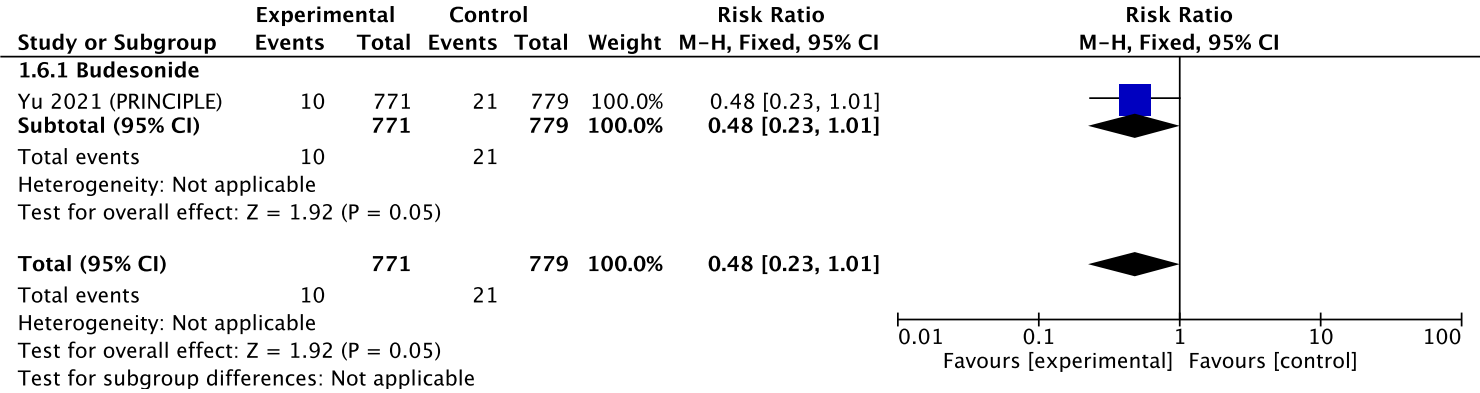

Supplement: S4 Fig — (PDF) [file pone.0294872.s005.pdf]

S6 mechanical ventilation

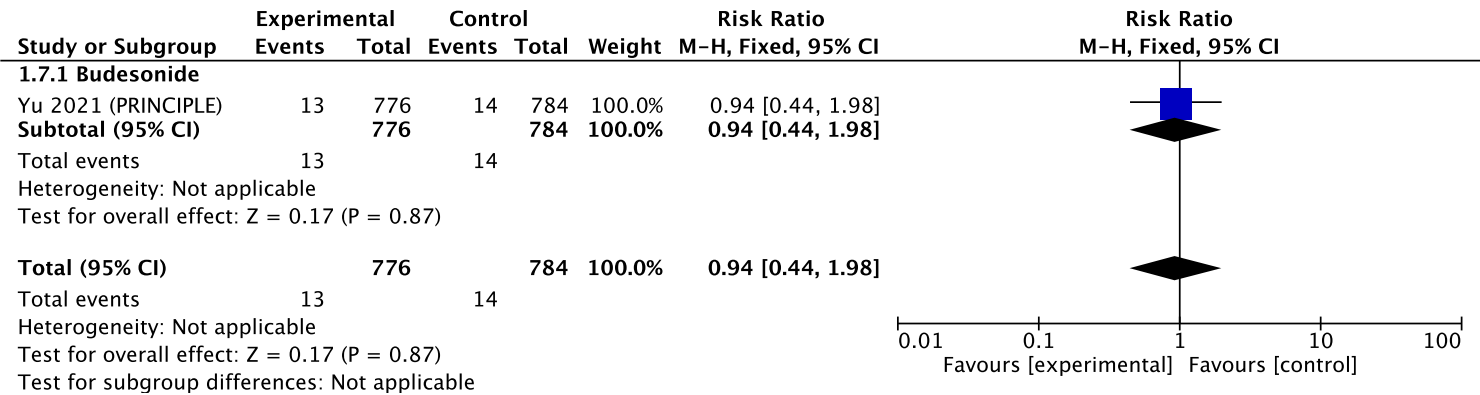

Supplement: S5 Fig — (PDF) [file pone.0294872.s006.pdf]
