# Supplementary material for: Clinical efficacy of inhaled corticosteroids in patients with coronavirus disease 2019: A living review and meta-analysis
Source: PLoS One. 2023 Nov 28;18(11):e0294872. doi: 10.1371/journal.pone.0294872 (PMC10684004; doi:10.1371/journal.pone.0294872)
Supplement: S6 Fig — Clinical Recovery by comparator (A, B). (PDF) [file pone.0294872.s007.pdf]

S7(A) Clinical Recovery at 7 days by comparator

Study or Subgroup	Experimental Events	Experimental Total	Control Events	Control Total	Weight	Risk Ratio M-H, Random, 95% CI
**2.4.2 Placebo**						
Clemency 2021	28	197	29	203	9.1%	0.99 [0.62, 1.61]
Ezer 2021 (CONTAIN)	25	105	22	98	8.3%	1.06 [0.64, 1.75]
**Subtotal (95% CI)**		**302**		**301**	**17.3%**	**1.03 [0.72, 1.45]**
Total events	53		51			
Heterogeneity: $Tau^2 = 0.00$; $Chi^2 = 0.03$, df = 1 (P = 0.86); $I^2 = 0\%$						
Test for overall effect: Z = 0.14 (P = 0.89)						
**2.4.3 No placebo**						
Duvignaud 2022 (COVERAGE)	37	107	31	104	13.5%	1.16 [0.78, 1.72]
Ramakrishnan 2021 (STOIC) (1)	36	66	30	64	18.0%	1.16 [0.83, 1.64]
Song 2021	19	35	15	26	10.4%	0.94 [0.60, 1.47]
Yu 2021 (PRINCIPLE)	149	787	109	799	40.8%	1.39 [1.11, 1.74]
**Subtotal (95% CI)**		**995**		**993**	**82.7%**	**1.24 [1.05, 1.45]**
Total events	241		185			
Heterogeneity: $Tau^2 = 0.00$; $Chi^2 = 2.78$, df = 3 (P = 0.43); $I^2 = 0\%$						
Test for overall effect: Z = 2.60 (P = 0.009)						
**Total (95% CI)**		**1297**		**1294**	**100.0%**	**1.20 [1.03, 1.38]**
Total events	294		236			
Heterogeneity: $Tau^2 = 0.00$; $Chi^2 = 3.65$, df = 5 (P = 0.60); $I^2 = 0\%$						
Test for overall effect: Z = 2.42 (P = 0.02)						
Test for subgroup differences: $Chi^2 = 0.91$, df = 1 (P = 0.34), $I^2 = 0\%$						

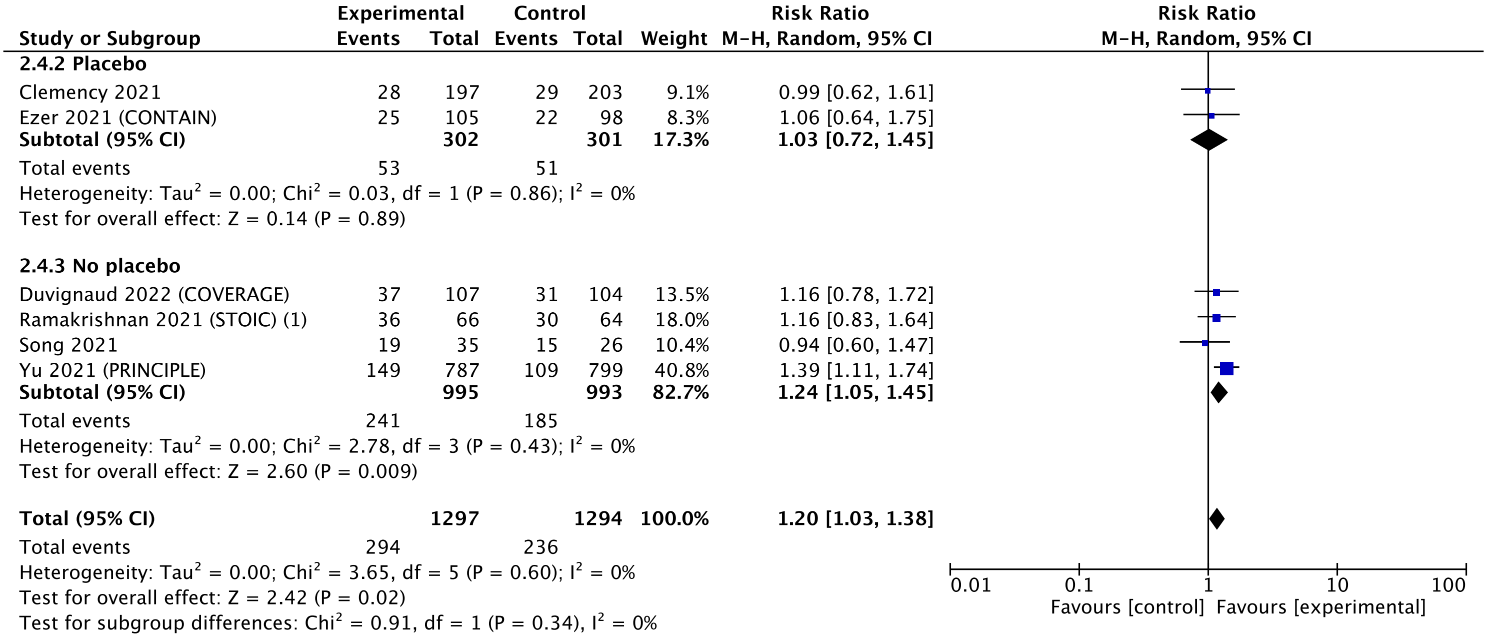

Footnotes
(1) At day 8, only those with confirmed COVID 19 infection

S7(A) Clinical Recovery at 7 days by comparator

Study or Subgroup	Experimental Events	Experimental Total	Control Events	Control Total	Weight	Risk Ratio M-H, Random, 95% CI
**2.3.2 Placebo**						
Clemency 2021	81	197	76	203	15.5%	1.10 [0.86, 1.40]
Ezer 2021 (CONTAIN)	57	105	44	98	12.7%	1.21 [0.91, 1.60]
**Subtotal (95% CI)**		**302**		**301**	**28.3%**	**1.14 [0.95, 1.38]**
Total events	138		120			
Heterogeneity: $Tau^2 = 0.00$; $Chi^2 = 0.26$, df = 1 (P = 0.61); $I^2 = 0\%$						
Test for overall effect: Z = 1.44 (P = 0.15)						
**2.3.3 No placebo**						
Duvignaud 2022 (COVERAGE)	57	105	57	100	15.5%	0.95 [0.75, 1.22]
Ramakrishnan 2021 (STOIC) (1)	60	66	46	64	23.6%	1.26 [1.07, 1.50]
Song 2021	26	35	14	26	7.1%	1.38 [0.92, 2.07]
Yu 2021 (PRINCIPLE)	263	787	192	799	25.5%	1.39 [1.19, 1.63]
**Subtotal (95% CI)**		**993**		**989**	**71.7%**	**1.23 [1.04, 1.46]**
Total events	406		309			
Heterogeneity: $Tau^2 = 0.02$; $Chi^2 = 6.93$, df = 3 (P = 0.07); $I^2 = 57\%$						
Test for overall effect: Z = 2.47 (P = 0.01)						
**Total (95% CI)**		**1295**		**1290**	**100.0%**	**1.21 [1.08, 1.36]**
Total events	544		429			
Heterogeneity: $Tau^2 = 0.01$; $Chi^2 = 7.86$, df = 5 (P = 0.16); $I^2 = 36\%$						
Test for overall effect: Z = 3.24 (P = 0.001)						
Test for subgroup differences: $Chi^2 = 0.35$, df = 1 (P = 0.55), $I^2 = 0\%$						

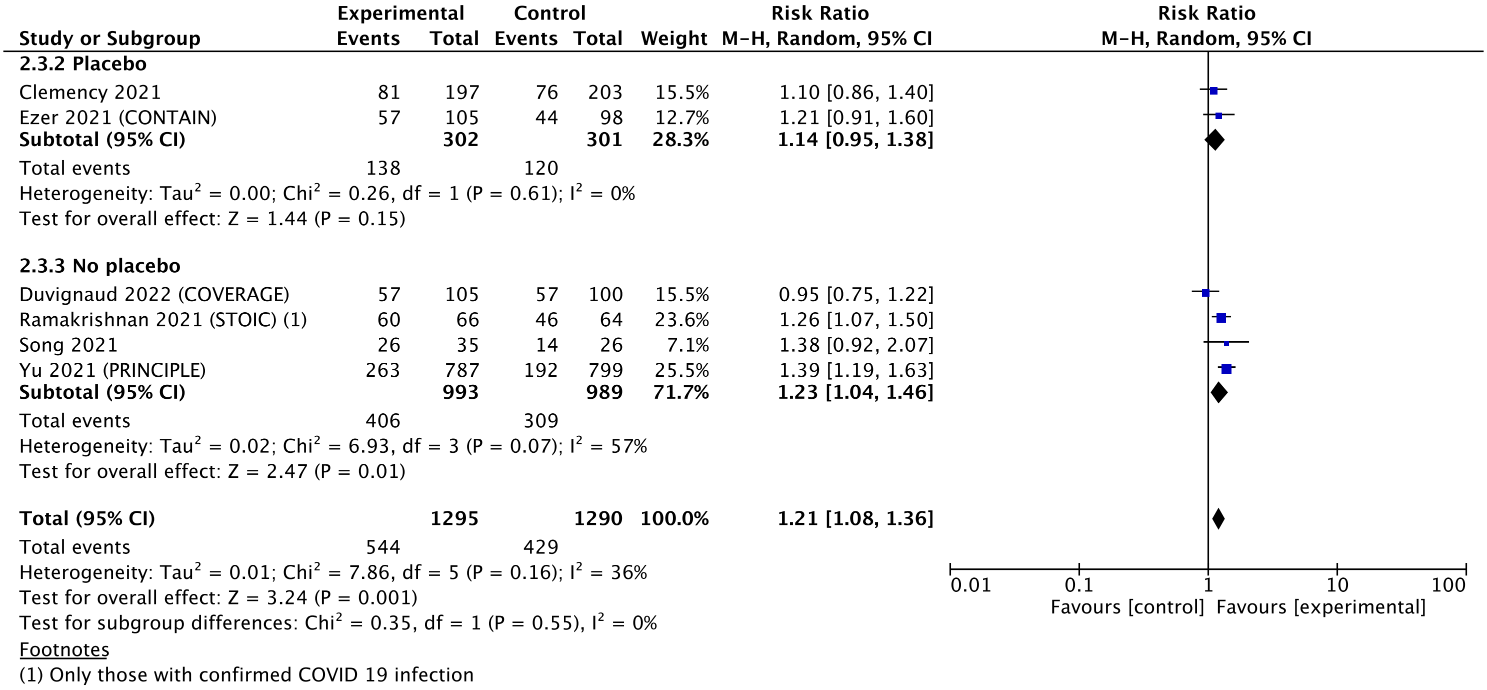

Footnotes
(1) Only those with confirmed COVID 19 infection
